# Development of a stem cell spheroid‐laden patch with high retention at skin wound site

**DOI:** 10.1002/btm2.10279

**Published:** 2021-12-28

**Authors:** Gun‐Jae Jeong, Gwang‐Bum Im, Tae‐Jin Lee, Sung‐Won Kim, Hye Ran Jeon, Dong‐Hyun Lee, Sangyul Baik, Changhyun Pang, Tae‐Hyung Kim, Dong‐Ik Kim, Young Charles Jang, Suk Ho Bhang

**Affiliations:** ^1^ School of Biological Sciences Georgia Institute of Technology Atlanta Georgia USA; ^2^ School of Chemical Engineering Sungkyunkwan University Suwon South Korea; ^3^ Department of Bio‐Health Convergence Kangwon National University Chuncheon Gwangwon South Korea; ^4^ Department of Medical Biotechnology, School of Biomedical Science Kangwon National University Chuncheon South Korea; ^5^ Division of Vascular Surgery, Samsung Medical Center School of Medicine, Sungkyunkwan University Seoul South Korea; ^6^ School of Integrative Engineering Chung‐Ang University Seoul South Korea

**Keywords:** 3D‐structured patch, adipose‐derived stem cell, angiogenesis, skin wound healing, spheroid

## Abstract

Mesenchymal stem cells such as human adipose tissue‐derived stem cells (hADSCs) have been used as a representative therapeutic agent for tissue regeneration because of their high proliferation and paracrine factor‐secreting abilities. However, certain points regarding conventional ADSC delivery systems, such as low cell density, secreted cytokine levels, and cell viability, still need to be addressed for treating severe wounds. In this study, we developed a three‐dimensional (3D) cavity‐structured stem cell‐laden system for overdense delivery of cells into severe wound sites. Our system includes a hydrophobic surface and cavities that can enhance the efficiency of cell delivery to the wound site. In particular, the cavities in the system facilitate hADSC spheroid formation, increasing therapeutic growth factor expression compared with 2D cultured cells. Our hADSC spheroid‐loaded patch exhibited remarkably improved cell localization at the wound site and dramatic therapeutic efficacy compared to the conventional cell injection method. Taken together, the hADSC spheroid delivery system focused on cell delivery, and stem cell homing effect at the wound site showed a significantly enhanced wound healing effect. By overcoming the limitations of conventional cell delivery methods, our overdense cell delivery system can contribute to biomedical and clinical applications.

**Abbreviations**
3DP: 3D cavity‐structured patch
CLP: stem cell‐laden 3D cavity‐structured patch
EB: ethidium bromide
FDA: fluorescein diacetate
hADSC: human adipose tissue‐derived mesenchymal stem cell
MSC: mesenchymal stem cell
PBS: phosphate‐buffered saline
PDMS: polydimethylsiloxane
qRT‐PCR: quantitative reverse transcription‐polymerase chain reaction

## 1 INTRODUCTION

Mesenchymal stem cells (MSCs) have been widely utilized in recent years for regenerative medicine applications, including tissues such as bone, blood vessels, heart, and skin.^1^, ^2^, ^3^ Among the MSCs, human adipose tissue‐derived MSCs (hADSCs) have been highlighted as most suitable candidate for therapeutic applications because of their high proliferative potential, exosome secretion, autocrine and paracrine factors, and adipose tissue accessibility as a cell source.^4^, ^5^ Despite their strong potential as a donor cell source for stem cell‐based therapy, ADSCs implanted into lesions such as ulcers, ischemia, and wound areas have shown limited therapeutic efficacy due to their low survival rate at the wound site.^6^, ^7^ The major reason for this low survival rate is due to the harsh microenvironment at the wound site, caused by hypoxia, lack of nutrients, proinflammation, and loss of cell adhesion. To improve the engraftment of ADSCs in the wound area, several strategies have been developed. These strategies include the genetic modification of stem cells,^8^ delivery of stem cells with growth factors,^9^ use of tissue engineering scaffolds,^10^ and delivery of three‐dimensional (3D) stem cell cluster/aggregate.^11^ Although these strategies showed improvements at certain degrees, more efficient approaches are still required for the treatment of severe wounds.

Conventional treatments are focused on the management of skin wounds, which include removing necrotic tissue and reducing infection by replacing wound dressings and applying different kinds of ointments.^12^ However, for hard‐to‐heal wounds, including chronic wounds, major burn injuries (>20% of total body surface area burned), and full‐thickness severe skin wounds, comprehensive treatment is required rather than management.^13^ Recent advances in severe skin wound regeneration are mostly based on biomedical engineering approaches, such as stem cell delivery, cell‐free scaffolds, cellular skin substitutes, and 3D bioprinting.^14^, ^15^, ^16^ In particular, the stem cell delivery method for wound healing treatment is a promising method because of its immune regulatory function and regenerative factor secretion. Although stem cells (including ADSCs) have great potential for the treatment of severe wounds, enhanced and efficient stem cell delivery methods are necessary for clinical treatment.

To overcome some of the limitations of previous studies, we developed a 3D cavity‐structured cell‐laden system for overdense delivery of cells into severe wound sites. The 3D structure of the patch was designed to simplify the formulation of ADSC spheroids. We fabricated a hydrophobic surface patch and many cavities to enhance the number of loaded cells into the patch, which complemented the weakness of the conventional patch system and low delivery density of cells. The ADSC spheroids in the patch had an increased expression of therapeutic growth factors compared to 2D cultured cells in vitro. Previous reports have shown that the increasing diameter of 3D cell aggregate can lead the upregulation of hypoxia inducible factor 1 alpha (HIF‐1α) and HIF‐1α‐related angiogenic factors expression.^17^, ^18^, ^19^ Based on the enhanced therapeutic gene expression from 3D cell aggregate, various attempted to treat intractable diseases with 3D cell aggregates have been developed. Recent report has shown that the angiogenic paracrine factor secretion from 3D cell aggregate, spheroid, can be optimized by controlling cell culture period and diameter range.^17^ Moreover, combining the spheroids with new materials such as hydrogel to enhance the therapeutic efficacy of the cell has been reported.^18^ However, conventional methods required time consuming and labor‐intensive procedure for preparing the spheroids. Also, injecting spheroids with syringes can induce shear stress to the spheroids during the injection process. The stress leads low cell viability and structural damage that can decrease the therapeutic effect of spheroid. Therefore, to increase the therapeutic efficacy of spheroids, it is necessary to develop easy and fast spheroid transplantation method without structural damage. Here, we have suggested a patch with a specific 3D structure that can induce and include spheroid easily and quickly. Moreover, our patch was designed to deliver spheroids directly to the wound sites without cellular or structural damages. When the hADSCs were applied with the 3D‐structured patch, the cells were localized in the wound site and had enhanced therapeutic efficacy compared to the conventional cell injection method. Particularly, we focused on the cellular efficacy of delivery and homing to the wound site, which illustrated the increased efficacy of our patch system. Our overdense cell delivery system represents an effective alternative to conventional cell injection methods and thus, could contribute to biomedical and clinical applications.

## 2 MATERIALS AND METHODS

### 2.1 Cell culture

**FIGURE 1:**
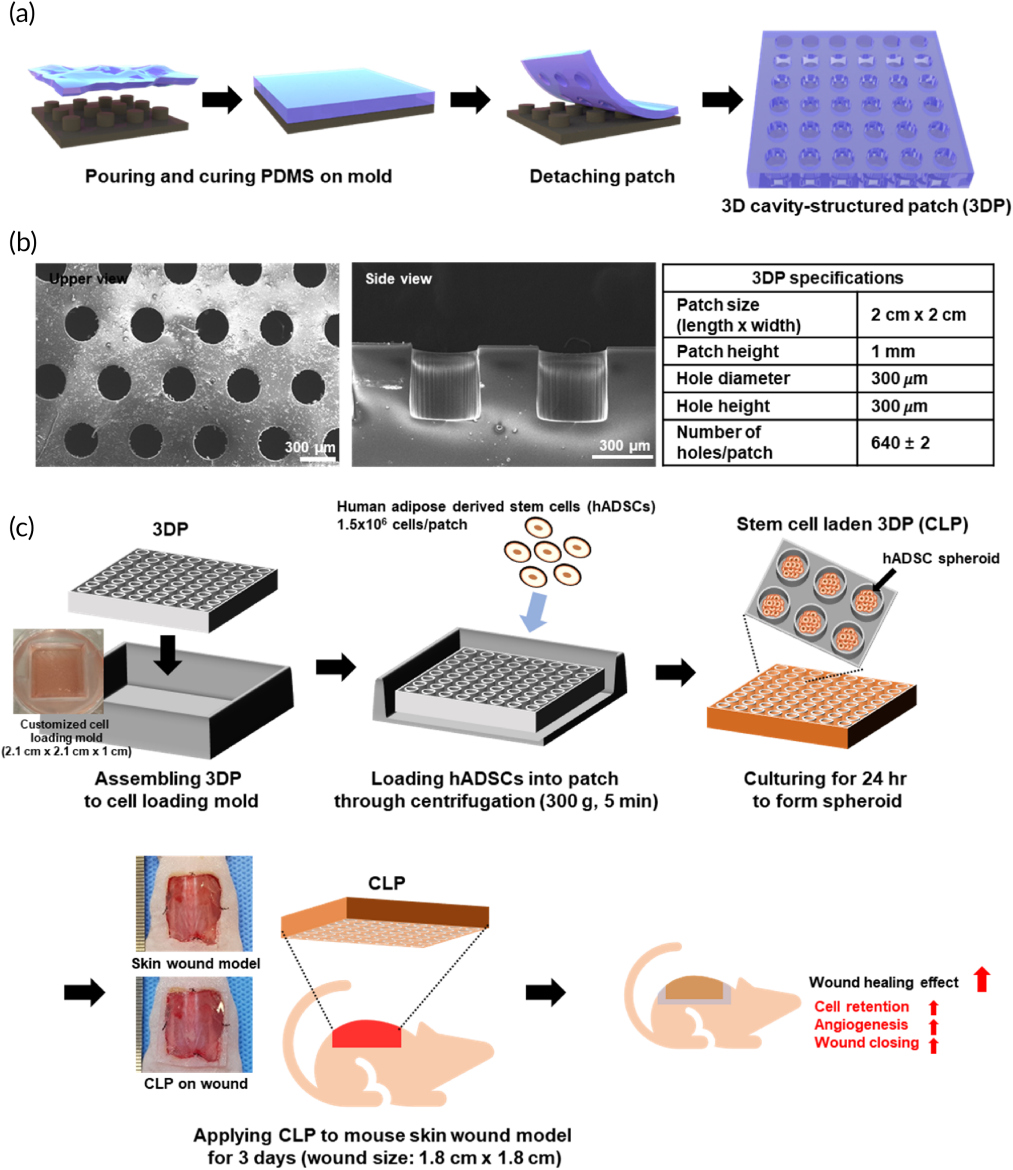
A schematic diagram of 3DP fabrication, CLP preparation, and in vivo experiments. (a) A schematic representation of 3DP fabrication. (b) Scanning electron microscopy image of fabricated 3DP and its structural specification. (c) Sequential diagram for CLP preparation and in vivo experiments. 3DP was assembled to customize cell‐loading mold. ADSCs were loaded onto patch by centrifugation. After incubation for 24 h, ADSCs formed spheroids in 3DP (CLP for in vivo experiment). The developed CLPs were subsequently applied to mouse skin wound model for 3 days after skin wound modeling. 3DP, 3D cavity‐structured patch; ADSC, adipose tissue‐derived mesenchymal stem cell; CLP, stem cell‐laden 3D cavity‐structured patch

The hADSCs were purchased from Lonza and maintained in Dulbecco's modified Eagle medium (Invitrogen) supplemented with 10% (vol/vol) fetal bovine serum (Gibco BRL) and 1% (vol/vol) antibiotics (penicillin–streptomycin; Gibco BRL) in a 5% (vol/vol) CO_2_ incubator at 37°C. hADSCs at passages 4–6 were used for the experiments. To load cells into the 3D cavity‐structured patches (3DPs), a cell‐loading mold was designed and fabricated in six‐well plates (Corning). A square pit [2.1 (W) × 2.1 (L) × 1 (H) cm] was made with polydimethylsiloxane (PDMS) in one of the wells of the six‐well plate. The size of the mold was determined to reduce cell loss during the stem cell‐laden 3D cavity‐structured patch (CLP) preparation, as shown in Figure 1c. After the insertion of 3DP into the cell‐loading mold, the plate was filled with sterile phosphate‐buffered saline (PBS; Gibco BRL) and centrifuged (400*g*, 5 min) to remove any bubbles in the 3DP cavities. PBS in the cell plate was subsequently removed, and detached ADSCs were loaded into the 3DP cavities by centrifugation (300*g*, 5 min for 1.5 × 10^6^ cells per patch). After centrifugation, the plate was incubated for 24 h in a 5% (vol/vol) CO_2_ incubator at 37°C.

### 2.2 Cell viability assay

Live and dead cells were imaged after staining with fluorescein diacetate (FDA; Sigma) and ethidium bromide (EB; Sigma). Dead cells were stained red because of the nuclear permeability of EB. Viable cells, which can convert the nonfluorescent FDA into fluorescein, were stained green. To compare the cell viability of the 2D surface and 3DP, identically sized PDMS compared to the 3DP patch without a 3D structure (flat PDMS) was prepared. Next, the same number of cells applied to the CLP was loaded onto the flat PDMS. The cells were stained with FDA and EB following a 24‐h incubation.

### 2.3 Wound treatment

Eight‐week‐old male athymic mice (20–25 g body weight; Orient) were anesthetized by inhalation of 2.5% isoflurane in oxygen gas. The skin wound area was marked using a 1.8 × 1.8 cm square shaped stamp on the back of each mouse. The epidermis, dermis, and stratum corneum in the marked areas were surgically removed. After skin incision and removal, four surgical sutures (6–0 silk suture; AILEE Co.) were made at the center of each side of the square‐shaped wound to prevent skin contracture.^20^ Immediately after skin wound modeling, the mice were randomly divided into four groups: (1) no treatment (skin wound covered with commercial skin dressing, Tegaderm [3M Healthcare]); (2) 3DP (skin wound covered with 3DP without cells); (3) cell injection (1.5 × 10^6^ hADSCs in 200 μl; PBS; 50 μl cell suspension was intradermally injected to each side of the square wound); and (4) CLP (skin wound covered with a 1.5 × 10^6^ hADSC‐loaded CLP). Tegaderm skin dressings were replaced at Days 3, 5, 10, and 14 after inducing wound modeling. For the 3DP and CLP groups, the PDMS patches were removed 3 days after treatment, and skin dressing (Tegaderm) was applied. The untreated group served as the control group. All animals received care according to the Guidelines for the Care and Use of Laboratory Animals of Sungkyunkwan University (SKKUIACUC2020‐01‐12‐1, January 2020).

### 2.4 In vivo wound imaging and wound closure quantification

The wounds were photographed at each time point. Wound closure was quantified and calculated based on the pixel‐to‐centimeter ratio. The wound size was measured using an imaging software (ImageJ). The wound margin was defined as a grossly visible margin of epithelial tissue migration toward the center of the wound and over the granulation tissue bed. Wound closure was calculated as the percentage of the initial wound area [(wound area at time point)/(initial wound area) × 100%].^21^

### 2.5 Live imaging of transplanted cells

To evaluate hADSC engraftment and retention after treatment, hADSCs were labeled with VivoTrack 680 (PerkinElmer) or DiI (Thermo Fisher Scientific) and injected or delivered with CLP at the wound site. Luminescence intensity was monitored (the images were taken after Days 0, 1, 2, 3, 4, 9, and 14 days after surgery and each treatment) and quantified for 14 days using an IVIS Spectrum Live Imaging System (PerkinElmer).

### 2.6 Quantitative reverse transcription‐polymerase chain reaction

Quantitative reverse transcription‐polymerase chain reaction (qRT‐PCR) analysis was used to quantify relative messenger RNA expression. Total RNA was extracted from samples (*n* = 4 per group) using 1 mL TRIzol reagent (Invitrogen) and 200 μl chloroform. The lysed samples were centrifuged at 12,000 rpm for 10 min at 4°C. The RNA pellet was washed with 75% (vol/vol) ethanol in water and dried. The samples were subsequently dissolved in RNase‐free water. For qRT‐PCR, SYBR Green Real‐Time PCR Master Mix (Thermo Fisher Scientific) was used. β‐actin was used as the internal control.

### 2.7 Statistical analysis

Quantitative data are expressed as the mean ± *SD*. Prism 9 software (GraphPad Software) was used to perform one‐way analysis of variance. Tukey's test was performed when significant differences were detected. *p* < 0.05 were considered as statistically significant. Data shown in this study are from a representative experiment repeated three times with similar results.

## 3 RESULTS

### 3.1 3DP fabrication and characterization and CLP preparation

In our previous research, MSC spheroids (150–200 μm) showed enhanced angiogenic and regenerative potential compared to 2D cultured cells. Based on previous results, a 300‐μm diameter cavity structure was selected for the formation of hADSC spheroids. 3DPs were prepared by stamping PDMS from a negatively patterned silicon wafer (Figure 1a). The 3DP patch had 640 cavities and a patch size of 2 × 2 cm (Figure 1b). After sterilization, 3DPs were assembled into a customized cell‐loading mold comprising PDMS and a square pit [2.1 (W) × 2.1(L) × 1 (H) cm] in a well of a six‐well plate. Next, 1.5 × 10^6^ cells per patch were loaded into 3DP by centrifugation and cultured for 24 h in a conventional cell culture incubator. After 24 h of patch culture, CLP was prepared and applied to the mouse skin wound model (Figure 1c).

### 3.2 In vitro characterization of CLP

**FIGURE 2:**
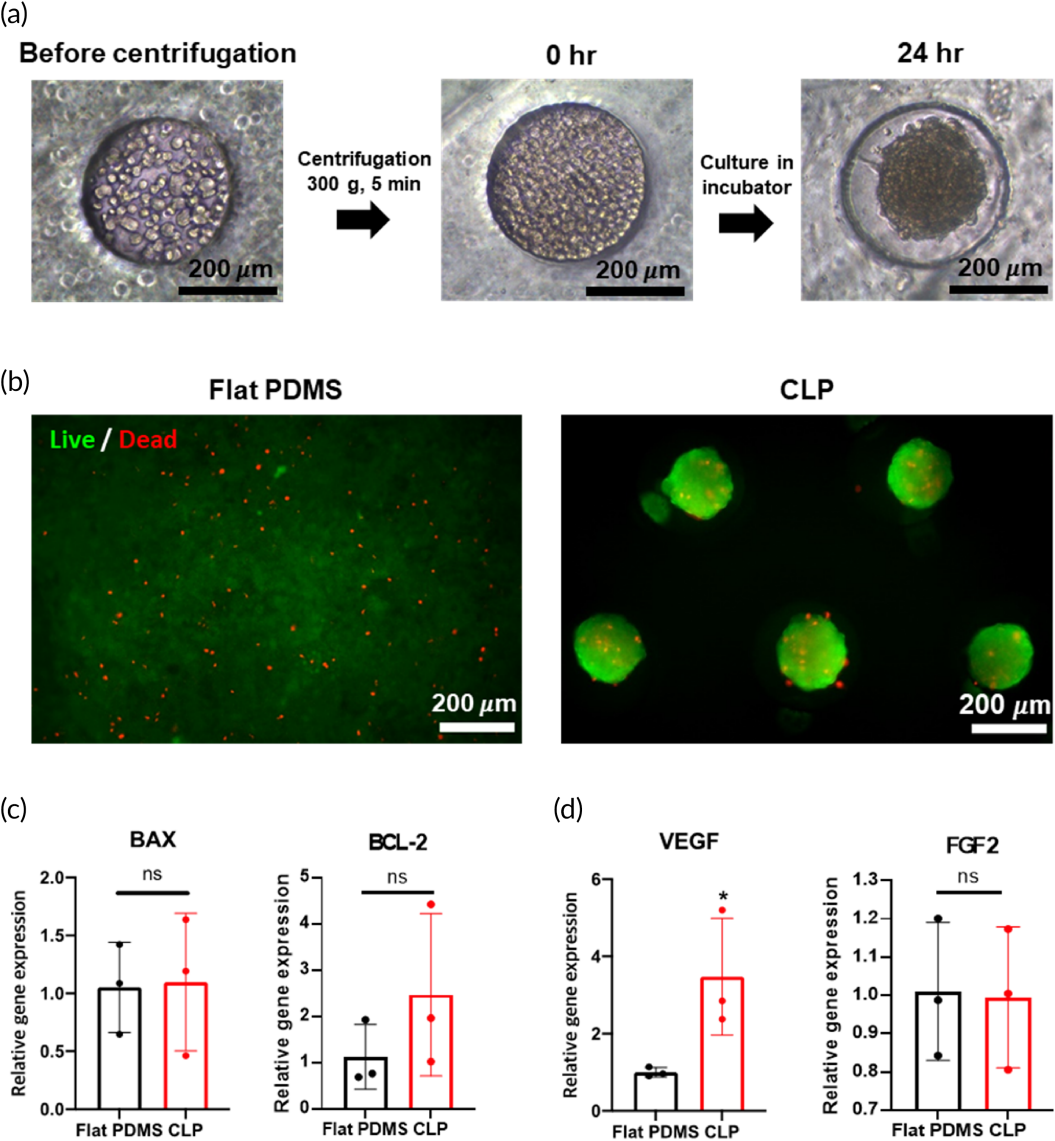
ADSC spheroid formation and CLP characterization. (a) Bright field images for CLP preparation. Before centrifugation, cells were dispersed inside and outside of cavities in CLP. By centrifugation, cells filled the cavities and formed spheroids after incubation for 24 h. (b) Cell viability assay after loading and 24‐h incubation. Flat PDMS (PDMS patch without 3D structure) group was prepared with identical cell‐loading procedure of CLP. (c) Gene expression level of proapoptotic (*BAX*) and antiapoptotic (*BCL2*) factors. (d) The gene expression levels of angiogenic factors (*VEGF* and *FGF2*). *p < 0.05 as compared with flat PDMS group. ADSC, adipose tissue‐derived mesenchymal stem cell; CLP, stem cell‐laden 3D cavity‐structured patch; PDMS, polydimethylsiloxane

To examine spheroid formation in CLP, 1.5 × 10^6^ ADSCs were loaded onto 3DP (Figure 2a). Immediately after cell loading, most of the cells were placed in the 3D cavity structure of 3DP at 0 h. After 24 h of incubation, cells loaded in the 3DP structure formed spheroids. The diameter of the spheroids in CLP was 200 μm. Since a high density of cells can reduce cell viability, it was evaluated using live/dead assay and qRT‐PCR (*BAX* and *BCL2*, Figure 2b,c). The control group comprised 1.5 × 10^6^ hADSCs loaded on PDMS patches without a 3D cavity structure (flat PDMS). There was no significant difference in *BAX* and *BCL2* gene expression levels between the flat PDMS and CLP groups (Figure 2c). To investigate regenerative factor expression in the CLP group, *VEGF* and *FGF2* gene expression levels were examined (Figure 2d). Compared with the flat PDMS group, *VEGF* gene expression levels were significantly higher in the CLP group. However, *FGF2* gene expression levels were not significantly different between the two groups.

### 3.3 In vivo stem cell delivery after CLP treatment

**FIGURE 3:**
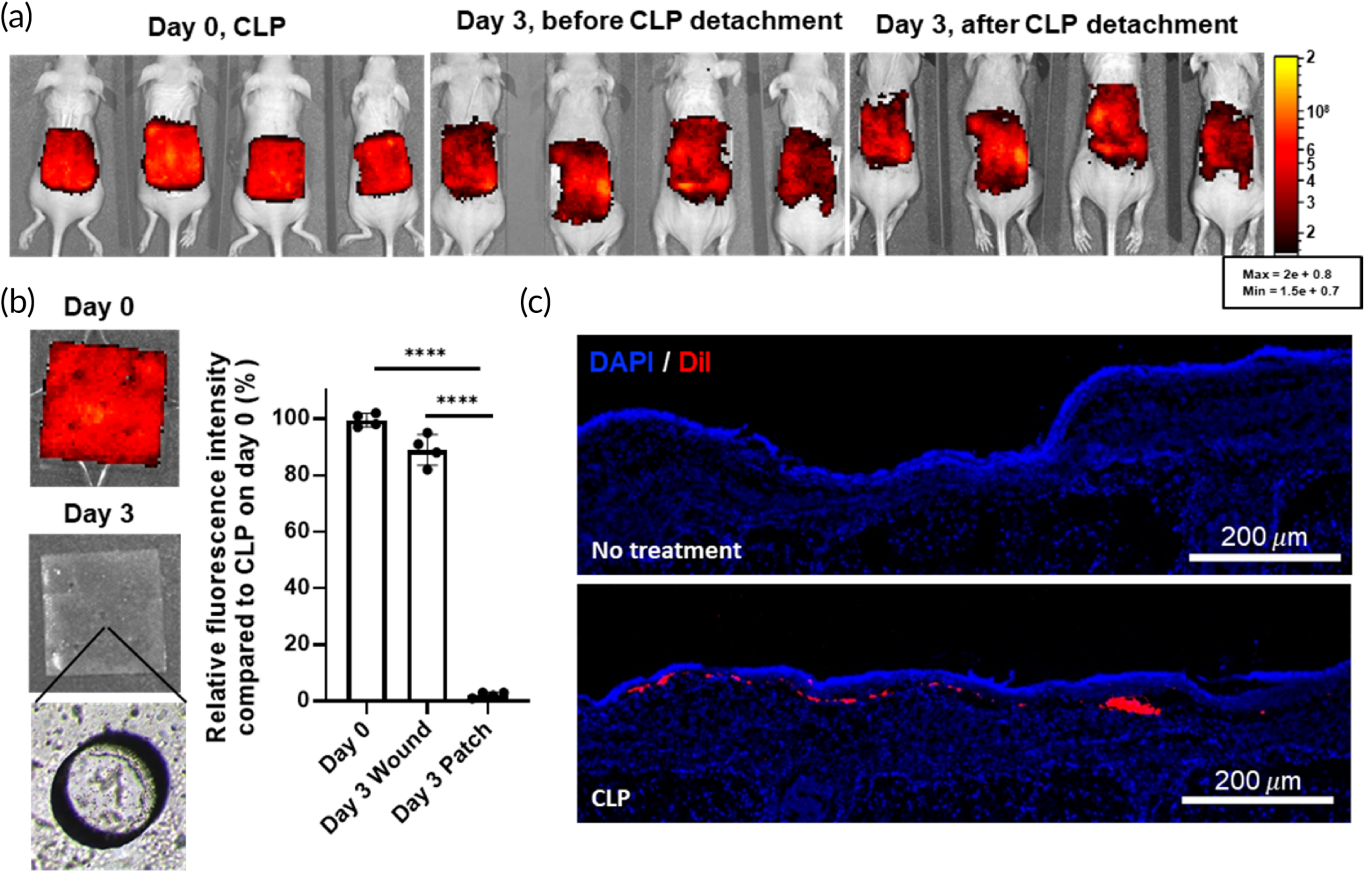
hADSC delivery to skin wound site 3 days after CLP administration. (a) in vivo live imaging of VivoTrack 680‐stained hADSCs on Days 0 and 3 before and after CLP detachment. (b) Imaging of fluorescent labeled ADSCs on Days 0 and 3 after CLP detachment. Left panel shows representative images of CLP on Days 0 and 3. Bright field image (bottom left panel) shows emptied cavities in CLP. The fluorescence intensities of CLP on Days 0 and 3 before and after patch detachment were quantified (right panel). ****p < 0.0001 compared to CLP on Day 0. (c) DiI (red) labeled hADSCs were loaded onto CLP and delivered to skin wound site. Samples were prepared 3 days after skin wound modeling and treatment. Cell nuclei were stained with DAPI (blue). CLP, stem cell‐laden 3D cavity‐structured patch; hADSC, human adipose tissue‐derived mesenchymal stem cell

In vivo live imaging displayed that most of the cells in the patch were transferred to the wound site without leakage to other sites after CLP detachment compared to other groups (Figure 3a). The quantification data of fluorescent intensity from Day 0 CLP, Day 3 wound site, and Day 3 CLP after detachment indicated that most of the hADSCs in the CLP patch had migrated to the wound site (Figure 3b). After 3 days, only 2.25 ± 0.95% of cells remained in the patch, as shown in representative fluorescent images of the patch (Figure 3b, Day 3 bottom panel). Cross‐sectional images from the wound site on Day 3 showed that hADSCs were successfully delivered to the wound sites (Figure 3c).

### 3.4 Deposition of delivered stem cells after CLP treatment

**FIGURE 4:**
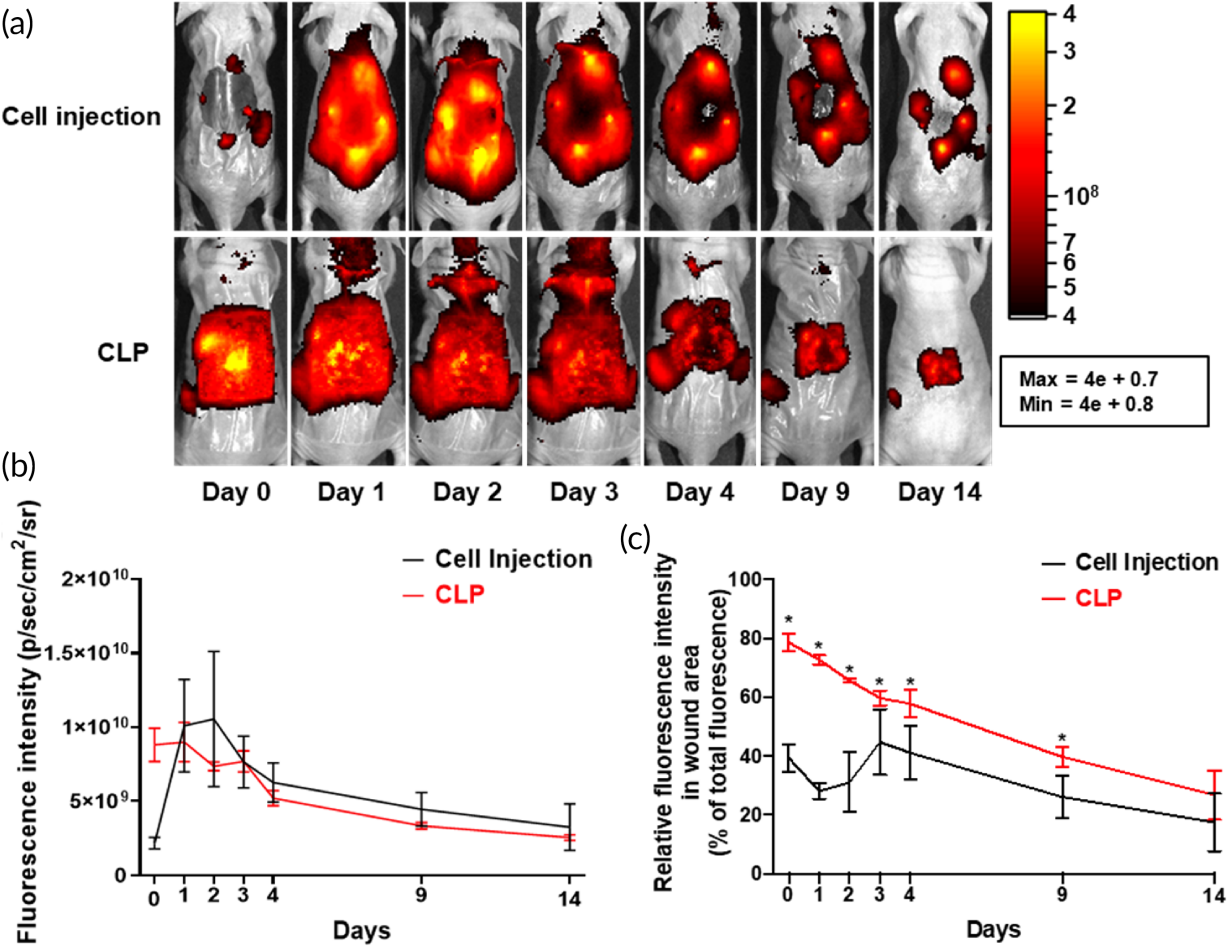
Upregulated CLP‐delivered cell retention at wound site. (a) Live imaging of hADSCs in cell injection and CLP groups from Days 0 to 14. CLP was removed on Day 3. (b) Quantification of relative fluorescence intensity of each group (*n* = 6 per group). (c) Relative fluorescence intensity in wound area based on the total fluorescence in mouse. *p < 0.05 as compared with cell injection group (*n* = 6 per group). CLP, stem cell‐laden 3D cavity‐structured patch

The deposition of delivered stem cells was examined using in vivo live imaging (Figure 4). VivoTrack 680 labeled 1.5 × 10^6^ hADSCs were loaded onto the CLP and delivered to the wound model on Day 0 (CLP group). For the control group, 1.5 × 10^6^ hADSCs were prepared in 200 μl PBS and injected at each side of the wound (50 μl for each of the four sides) on Day 0 (cell injection group). After delivering hADSCs with CLP and injection, fluorescence images were obtained from Days 0 to 14 (Figure 4a). The quantification data of total fluorescence intensity were not significantly different between the cell injection and CLP groups (Figure 4b). However, relative fluorescence intensity in the wound area was significantly higher in the CLP group than in the cell injection group until 9 days after treatment (Figure 4c). Higher fluorescence intensity at the wound site indicated that a greater number of cells remained in the wound site in the CLP group than in the cell injection group.

### 3.5 Enhanced skin wound healing after CLP treatment

**FIGURE 5:**
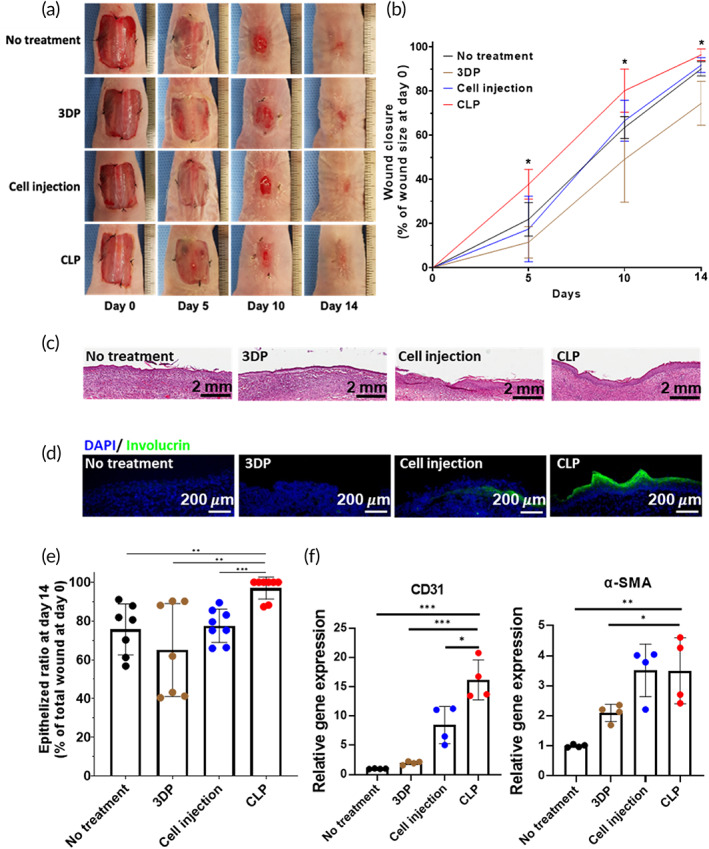
Accelerated skin wound healing and improved epithelization after CLP treatment. (a) Representative images of skin wound on Days 0, 5, 10, and 14. (b) Quantification of wound closure for 14 days (*n* = 7 per group). *p < 0.05 compared with other groups. (c) Representative hematoxylin and eosin staining image for each group (14 days after the treatments). Scale bar = 2 mm. (d) Immunohistochemical staining for involucrin (green) at the center of skin wound site 14 days after the treatments. Scale bar = 200 μm. (e) Quantification of epithelized area ratio as the percentage of total wound area. Significant differences between two groups are indicated as **p < 0.01 or ***p < 0.001. (f) Relative expression of CD31 and α‐SMA. Significant differences between two groups are indicated as *p < 0.05, **p < 0.01, or ***p < 0.001. CLP, stem cell‐laden 3D cavity‐structured patch

CLPs were applied to an in vivo mouse skin wound model to investigate their therapeutic effect in vivo. No treatment (Tegaderm treatment for 14 days), 3DP (3DP without hADSCs treatment for 3 days followed by Tegaderm treatment for the remaining 11 days), and cell injection (1.5 × 10^6^ hADSCs injected on each side of the skin wound and Tegaderm treatment for 14 days) groups served as control groups. For the CLP group, CLPs were applied to the wound site for 3 days followed by Tegaderm treatment for the remaining 11 days. The CLP group showed a higher wound closure rate from Days 5 to 14 (Figure 5a,b). Hematoxylin and eosin (H&E) staining and quantification of the re‐epithelized wound ratio revealed enhanced skin wound regeneration in the CLP group (Figure 5c,e). To confirm epidermis (the outermost part of the skin) regeneration, immunohistochemistry with involucrin was performed. Similar to the H&E staining results, involucrin expression was much higher in the outermost part of the wound site in the CLP group than in the other groups (Figure 5d). In addition to histological results, CD31 gene expression was enhanced in the CLP group compared with other groups, whereas α‐SMA gene expression was significantly enhanced in the CLP group compared to NT and 3DP groups (Figure 5f).

## 4 DISCUSSION

Skin wounds are one of the most demanding medical issues worldwide.^22^ Severe skin wounds, including chronic and large wounds, are difficult to heal using conventional wound care methods.^12^ Therefore, cell‐based therapies utilizing keratinocytes and MSCs have been developed to treat severe wounds.^2^, ^4^, ^23^ Although recent studies have shown certain enhancement of wound healing by utilizing different kinds of cell delivery approaches, these strategies have shortcomings such as low cell viability and cell dispersion after delivery. In this study, we developed a CLP for the easy and efficient delivery of hADSCs for severe wound treatment (Figure 1).

The 3DPs were manufactured using a biocompatible material, PDMS. This material offered sufficient rigidity to maintain the 3D structure during cell seeding and delivery procedure and elasticity for applying the patch to the mouse wound model (dorsal part of mouse). However, PDMS is not the perfect material for the treatment of skin wound because of its limited gas permeability and hydrophobicity.^24^, ^25^ These properties are obstacles for wound healing. As shown in Figure 5b, the group administered only 3DP (PDMS patch‐only group) had delayed wound healing compared to the other groups. Despite this drawback, stem cell loading in the CLP exhibited enhanced therapeutic efficacy compared with the same amount of stem cell injection at the wound site (Figure 5). Because of the concave structure of 3DP, cell spheroids were formulated when the stem cells were cultured in 3DP for 24 h (Figure 2a). The size of the stem cell spheroids was uniform in the patch (~200 μm diameter), and spheroid formation did not affect cell viability in the patch (Figure 2b,c). Previously, stem cell spheroids have been shown to exhibit enhanced therapeutic paracrine secretion due to the environment in the spheroidal structure.^11^, ^26^ In addition, stem cell spheroids in CLP showed increased *VEGF* gene expression, which was induced by enhancement of HIF‐1α protein expression (Figures 2D and S1).

The CLPs were removed 3 days post administration at the wound site to minimize the intervention effect of the PDMS patch. Live in vivo imaging with fluorescent‐labeled hADSC‐loaded patches revealed that stem cell delivery using CLP occurred within 3 days of treatment (Figure 3). After removing the CLP on Day 3, most of the fluorescence signal remained at the wound site. In the wound site, enhanced expression of SDF‐1 alpha, a key factor for cell migration, induces the migration of injected cells to the wound site.^27^ Since control group showed low cell viability and growth factor secretion, CLP group had more enhancement in wound healing effect compared with the control group (Figure S1). Notably, because of the optical transparency of CLP, the fluorescence signals from the cells were not affected by the patch. Compared with the cell injection method, the CLP group did not display enhanced fluorescence intensity until 14 days of monitoring (Figure 4b). However, the relative fluorescence intensity at the wound site was significantly higher during wound regeneration (Figure 4c). Consecutive in vivo monitoring of the wound site provided information about the changes post cell delivery. This means that delivering cells using the injection method may not be effective because the delivered cells mostly remain outside or at the boarder of the wound site. Therefore, injected cells cannot participate in the wound healing process after a certain point. In contrast, cells delivered using the CLP were successfully localized at the wound site and remained localized until Day 14, suggesting that the delivered stem cells have a better chance to be involved in the wound healing process by this method compared to others. As a result of the effective delivery of hADSCs with CLP, the wound healing rate and vascularization markers were significantly increased. Furthermore, an establishment of well‐developed epithelial cell layer was confirmed using involucrin staining (Figure 5).^28^

## 5 CONCLUSION

In this study, we fabricated an overdensely CLP for the treatment of severe wounds. This stem cell delivery patch system can be easily transferred to the clinical setup for the treatment of chronic or slow‐healing wounds by extending the size of the cell patch. However, the material that we used (PDMS) should be substituted for skin wound favorable materials that have characteristics including gas and liquid permeability and biodegradability. To improve the wound healing effect, different cell types (e.g., keratinocytes and fibroblasts) can be codelivered with stem cells. Overall, the CLP developed in this study is an easy and effective method for the treatment of hard‐to‐heal wounds.

## AUTHOR CONTRIBUTIONS

**Gun‐Jae Jeong:** Conceptualization (equal); data curation (equal); formal analysis (equal); investigation (equal); methodology (equal); visualization (equal); writing – original draft (equal). **Gwang‐Bum Im:** Conceptualization (equal); data curation (equal); formal analysis (equal); investigation (equal); methodology (equal); visualization (equal); writing – original draft (equal). **Tae‐Jin Lee:** Conceptualization (equal). **Sung‐Won Kim:** Investigation (supporting). **Hye Ran Jeon:** Investigation (supporting). **Dong‐Hyun Lee:** Investigation (supporting). **Sang‐yul Baik:** Investigation (supporting). **Chang‐hyun Pang:** Writing – review and editing (supporting). **Tae‐Hyung Kim:** Writing – review and editing (supporting). **Young Charles Jang:** Investigation (supporting); methodology (supporting); writing – review and editing (supporting). **Dong‐Ik Kim:** Funding acquisition (equal); writing – review and editing (supporting). **Suk Ho Bhang:** Funding acquisition (equal); investigation (equal); project administration (equal); supervision (equal); writing – original draft (equal); writing – review and editing (equal).

## CONFLICT OF INTERESTS

The authors declare no conflicts of interests.

### PEER REVIEW

The peer review history for this article is available at https://publons.com/publon/10.1002/btm2.10279.

## Supporting information


**Appendix** S1: Supporting information

## Data Availability

Data available on request from the authors.
