# Supplementary material for: Development of a stem cell spheroid‐laden patch with high retention at skin wound site
Source: Bioeng Transl Med. 2021 Dec 28;7(2):e10279. doi: 10.1002/btm2.10279 (PMC9115685; doi:10.1002/btm2.10279)
Supplement: Supplementary file 1 — Appendix S1: Supporting information [file BTM2-7-e10279-s001.docx]

Supporting Information

**Development of a stem cell spheroid-laden patch with high retention at skin wound site**

Gun-Jae Jeong^a,#^, Gwang-Bum Im^b,#^, Tae-Jin Lee^c^, Sung-Won Kim^b^, Hye Ran Jeon^d^, Dong-Hyun Lee^b^, Sang-yul Baik^b^, Chang-hyun Pang^b^, Tae-Hyung Kim^e^, Dong-ik Kim^d^, Young Charles Jang^a^, Suk Ho Bhang^b,*^

^a^ School of Biological Sciences, Georgia Institute of Technology, Atlanta, GA 30332, USA

^b^ School of Chemical Engineering, Sungkyunkwan University, Suwon 16419, Republic of Korea

^c^ Department of Medical Biotechnology, Division of Medical Biotechnology, College of Biomedical Science, Kangwon National University, Chuncheon, 24341, Republic of Korea

^d^ Division of Vascular Surgery, Samsung Medical Center, Sungkyunkwan University, School of Medicine, Seoul 06351, Republic of Korea

^e^ School of Integrative Engineering, Chung-Ang University, Seoul, 06974, Republic of Korea

^#^ These authors contributed equally to this work

*** Corresponding author:** Suk Ho Bhang, Ph.D.,

E-mail: sukhobhang@skku.edu; Tel.: +82-31-290-7242; Fax: +82-31-290-7272

**Materials and methods**

*3D cavity-structured patch (3DP) fabrication and characterization*

The 3DP was prepared using a patterned polyurethane-acrylate mold and 5 wt% polydimethylsiloxane (PDMS) (Sylgard, Dow Corning Co., Midland, MI, USA). The negative-tone photoresist SU-8 was micropatterned on a silicon wafer using a photolithography process. A PDMS solution prepared from a mixture of PDMS base and curing agent (Sylgard, Dow Corning Co.) in the ratio of 20:1 was poured onto the customized mold. The PDMS was cured in an oven at 80°C for 2 h after removing the bubbles in a vacuum chamber. After detaching the cured PDMS patch from the mold, patches were trimmed to obtain square-shaped (2 × 2 cm) 3DPs (Figure 1A). The surfaces of the 3DPs were observed using a scanning electron microscope (SEM, JSM-6510, JEOL Ltd, Tokyo, Japan). The depth and size of the cavities in the 3DP were measured from the SEM images (Figure 1B).

*Histological examination*

Microscopic skin wound healing was observed by hematoxylin and eosin (H&E) staining of tissue sections using a light microscope (CKX53, Olympus, Tokyo, Japan). The skin tissue samples were fixed in paraformaldehyde, dehydrated with 30% sucrose, and embedded in an optimum cutting temperature (OCT) compound (SciGen Scientific, Gardenas, CA, USA). The specimens were then sliced into 10-µm thick sections and stained with H&E to examine skin wound healing.

*Immunohistochemistry*

For immunohistochemical staining, samples embedded in OCT compound were cut into 10-μm thick sections at −22°C. Immunohistochemistry analysis of epithelialized tissue was performed using an involucrin antibody (Abcam, Cambridge, UK). Involucrin was visualized with fluorescein isothiocyanate-conjugated secondary antibodies (Jackson Immuno Research Laboratories, West Grove, PA, USA). The sections were counterstained with 4′,6-diamidino-2-phenylindole (DAPI) and examined using fluorescence microscopy (DFC 3000 G, Leica).

*Western blot*

hADSCs were collected and lysed in radioimmunoprecipitation lysis buffer (Rockland Immunochemicals, Inc., Limerick, PA, USA). After centrifugation at 10,000 × g for 10 min, the supernatant was prepared as a protein extract. Protein concentrations were determined using bicinchoninic acid (BCA) protein assay (Pierce Biotechnology, Rockford, IL, USA). The same protein concentration was used in each sample, which was mixed with sample buffer, loaded, and subjected to sodium dodecyl sulfate-polyacrylamide gel electrophoresis (SDS-PAGE) using a 10% (v/v) resolving gel. Proteins separated using SDS-PAGE were transferred to an immune-blot polyvinylidene flouride membrane (Bio‐Rad) and then probed with antibodies against GAPDH, FGF2, CAPASE-9, and HIF-1α (R&D Systems, Minneapolis, MN, USA) overnight at 4 °C. They were then washed and incubated with horseradish peroxidase (HRP)‐conjugated secondary antibody (R&D Systems) for 1 h at room temperature. The blots were developed in a darkroom. Luminescence was recorded on an X-ray film in blue (Agfa HealthCare NV, Mortsel, Belgium). Bands were imaged using the Photoshop CC program (Adobe Systems, San Jose, CA, USA).


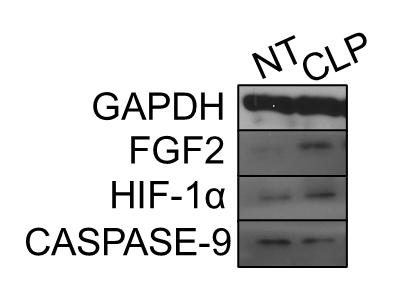


Figure S1. Protein expression of GAPDH, FGF2, HIF-1α, and CASPASE-9.
